# The influence of action–outcome contingency on motivation from control

**DOI:** 10.1007/s00221-018-5374-4

**Published:** 2018-09-14

**Authors:** Tegan Penton, Xingquan Wang, Michel-Pierre Coll, Caroline Catmur, Geoffrey Bird

**Affiliations:** 1 MRC Social, Genetic and Developmental Psychiatry Centre, Institute of Psychiatry, Psychology and Neuroscience, King’s College London, Denmark Hill, London, SE5 8AF UK; 2 Department of Experimental Psychology, University of Oxford, Oxford, OX1 3PH UK; 3 Department of Psychology, Institute of Psychiatry, Psychology and Neuroscience, King’s College London, Guy’s Campus, London, SE1 1UL UK

**Keywords:** Contingency, Motivation, Sense of agency, Individual differences

## Abstract

The sense of agency is defined as one’s sense of control over one’s actions and their consequences. A recent theory, the control-based response selection framework (Karsh and Eitam, Motivation from control: a response selection framework. The sense of agency, Oxford University Press, New York, 2015a), suggests that actions associated with a high sense of agency are intrinsically rewarding and thus motivate response selection. Previous studies support this theory by demonstrating that factors impacting on sense of agency (e.g. probability of an outcome following an action) also motivate selection of actions. Here we report a novel test of the control-based response selection framework in the domain of action–outcome contingency. The contingency between actions and their outcome has previously been demonstrated to impact the sense of agency, but its impact on the motivation to perform actions has not yet been examined. Participants were asked to press one of four buttons as randomly as possible. Each of the buttons was assigned a different probability of causing an outcome when pressed. Additionally, a contingency manipulation was employed where the probability of an outcome occurring in the absence of a button press was also varied in blocks throughout the experiment. Results demonstrated a significant influence of contingency on response speed, and a significant effect of probability on response selection, consistent with predictions from the control-based response selection framework. Furthermore, some evidence was observed for a positive correlation between influence of contingency and autistic traits, with individuals with higher autistic traits showing a greater influence of contingency on reaction times. The current findings support the idea that actions associated with an increased sense of agency are intrinsically rewarding, and identify how individual differences may impact on this process.

## Introduction

A sense of control over one’s own actions and their consequences (sense of agency) is an important component of self-awareness as it helps us determine our impact on the external world (Jeannerod 2003). This sense of agency has been linked with increased well-being (Ryan and Deci 2000; Welzel and Inglehart 2010) and has been shown to be impaired in various clinical conditions such as schizophrenia (Jeannerod 2003, 2009).

The sense of agency can be characterised by a pre-reflective feeling of agency (implicit sense of agency) usually measured by investigating differences in lower level sensory integration of a self-generated event and an externally generated event. Additionally, the sense of agency can be characterised by a reflective judgement of agency (explicit sense of agency) usually measured by investigating higher level evaluations of agency (Synofzik et al. 2008). Multiple factors have been shown to impact both the implicit and explicit sense of agency over an action and its consequence (e.g. see Moore and Obhi 2012, for review on factors influencing implicit sense of agency). For example, the extent to which an individual can predict the spatial and temporal characteristics, or form, of their action–outcome modulates the experience of implicit (spatial and temporal characteristics—Haggard et al. 2002; form—Moore and Haggard 2008) and explicit agency (spatial and temporal characteristics—Karsh et al. 2016). Additionally, the temporal delay between action and outcome influences the experience of agency, with smaller delays between action and outcome usually associated with an increased implicit (e.g. Blakemore et al. 1999, but also see; Humphreys and Buehner 2009; Wen et al. 2015) and explicit sense of agency (e.g. Sato and Yasuda 2005). One of the more extensively studied factors that influences our experience of agency is the probability of an outcome occurring following an action. Multiple studies have shown that increasing the likelihood of an outcome following an action increases the implicit (e.g. Engbert and Wohlschläger 2007; Moore and Haggard 2008; Voss et al. 2010) and explicit sense of agency (e.g. van der Weiden et al. 2011).

Whilst the *probability* of an outcome following an action is an important cue to agency, it is less of a veridical indicator of agency than the *contingency* between one’s actions and an outcome. If one considers only probability to determine one’s agency, then the fact that every time I clap my hands in a rainstorm, I am hit by a raindrop (i.e. the probability of a rain drop given a clap is 100%), would lead me to erroneously conclude that my clap causes the raindrop to hit me. In contrast, contingency refers to the difference in the probability of an outcome given that I act, and the probability of that outcome given that I do not act. In the rainstorm example, the probability of being hit by a raindrop when I clap is 100%, but the probability of being hit by a raindrop when I do not clap is also 100%, therefore, my clap is unlikely to be the cause of my being hit by a raindrop.

Indeed, there is a wealth of literature demonstrating the ability of humans to learn action–outcome contingencies (e.g. Allan and Jenkins 1980; Dickinson et al. 1984). More recently, Elsner and Hommel (2004) demonstrated that participants who experienced a learning phase in which a tone followed a keypress with a high degree of contingency, showed faster reaction times, in a subsequent test phase, to execute that keypress in response to the tone relative to a keypress not previously associated with the tone. The magnitude of this effect was greater for those who experienced a high keypress-tone contingency during the learning phase relative to those who experienced a lower degree of keypress-tone contingency during the learning phase. A parallel effect of the probability of the tone occurring on a given trial was observed even when contingency was held constant at zero (i.e. the probability of the tone given the keypress was equal to the probability of the tone in the absence of a keypress), such that participants who experienced a high overall probability of a tone occurring in the learning phase showed faster reaction times in response to the tone relative to a keypress not previously associated with the tone in a subsequent test phase. This effect was not observed for participants in lower probability conditions. Importantly, the perceived causality of the keypress in eliciting the tone (measured by explicit ratings) mirrored the response time effects. These findings demonstrate that both action–outcome contingency and the probability of an outcome can influence response speed and perceived causality. In addition to these data, several studies have demonstrated a relationship between action–outcome contingency and implicit sense of agency (e.g. Moore and Haggard 2008; Moore et al. 2009; Sidarus et al. 2013), demonstrating that action–outcome contingency, as well as the probability of outcome, influences the implicit sense of agency. Collectively, the existing research on contingency learning demonstrates that learned action–outcome contingency can influence response speed in the presence of the learned stimulus. However, the extent to which action–stimulus contingencies influence the degree to which participants are motivated to produce an action remains to be tested. The current study aims to address this gap in the literature. To do this, and in contrast to the previous work, the current paradigm will investigate the extent to which ‘being in control’ is motivating when (a) the action–outcome has not been previously associated with a specific response, (b) the action–outcome is task-irrelevant and (c) action–outcome contingency is manipulated on a within-subjects basis.

Addressing this gap in the literature is important for several reasons. One key area where this may be useful is in investigating individual differences in the sense of agency. Experience of agency, and the extent to which people privilege cues to agency, varies greatly among individuals and yet this issue is rarely investigated. To date, some of these individual differences have been explained by variance in personality traits such as schizotypy and attributional style (e.g. Moore and Bravin 2015; Penton et al. 2014), whereas more severe disturbances in the sense of agency are associated with clinical conditions such as schizophrenia (see Jeannerod 2009, for review) and to some extent, Autism Spectrum Disorder (see Zalla and Sperduti 2015, for review). Atypical use of cues to agency may impact on an individual’s sense of self and their self-awareness. As such, further investigation of individual differences in the experience of agency is an important area of study in both typical and atypical groups. One factor that may relate to individual differences in the experience of agency is the extent to which people are motivated by their sense of agency. Indeed, recent theoretical models and empirical work demonstrate that atypical use of agency cues may impact behaviour directly, causing individuals to be less likely to select instrumental (causal) actions over non-instrumental actions. The control-based response selection framework (Karsh and Eitam 2015a) posits that actions associated with an (explicit or implicit) sense of agency (control) are inherently rewarding, and are therefore likely to be selected (in the absence of any other criteria) over actions which do not result in a sense of agency. This framework has been tested in a series of studies by Karsh, Eitam and colleagues which show that those factors previously demonstrated to impact the sense of agency, such as probability of an outcome, and temporal contiguity and predictability of an outcome, also influence the likelihood of an action being selected (e.g. Karsh and Eitam 2015b; Karsh et al. 2016; Eitam et al. 2013). For example, in one study (Karsh and Eitam 2015b), three groups of participants (high probability group, mixed probability group, no effect group) were prompted to press one of four keys as randomly as possible when a white dot appeared on the screen. In the mixed probability group, each of the four buttons was assigned a different probability of an outcome occurring when they were pressed (0%, 30%, 60%, and 90% chance of outcome). In the high probability group, all buttons were assigned a high probability (90% chance of outcome) of the outcome occurring when pressed, whereas in the no effect group none of the buttons resulted in the outcome when pressed (0% chance of outcome). Although there were no differences in frequency or speed of presses across the four buttons in the high probability or no effect groups (although participants were faster overall in the high probability group compared to the no effect group), participants in the mixed probability group were faster and more likely to press buttons with a higher probability of an outcome than those with a lower probability of an outcome. The authors concluded that participants in the mixed probability condition were more motivated to engage in actions with a high probability of an outcome as they were associated with a greater sense of agency or control.

The current study seeks to test the Control-Based Response Selection Framework in a novel domain, that of action–outcome contingency, which could be considered to be the most valid cue to agency. Predictions from the Control-Based Response Selection Framework are clear; contingency manipulations should affect action responses as they have previously been demonstrated to impact upon the sense of agency (Moore and Haggard 2008; Moore et al. 2009; Sidarus et al. 2013). This prediction was tested using an adapted version of Karsh and Eitam’s (2015b) paradigm in which action–outcome contingency was manipulated. Accordingly, participants were prompted to press one of four keys as randomly as possible, and each of the four buttons was assigned a different probability of an outcome occurring when they were pressed (0%, 30%, 60%, and 90% chance of outcome). Across blocks of trials, the probability of the outcome occurring due to an external cause, irrespective of button presses, was also manipulated (0%, 30%, 60%, and 90% chance of an external outcome). The highest degree of contingency (0% chance of an external outcome) is comparable to the original Karsh and Eitam (2015b) procedure, and therefore one might expect the proportion of responses and reaction times to be influenced by the outcome probabilities assigned to the keys. At the lowest degree of contingency (90% chance of an external flash), the chances of an outcome occurring in the absence of a keypress are higher than in response to a keypress for three of the four buttons, and equiprobable for the fourth. If response selection and response speed are related solely to the probability of an outcome, then one would expect responses to be equally affected by outcome probability at all levels of contingency. In contrast, if contingency impacts agency and therefore response selection and response speed, one would expect to see a reduction in the effect of probability on response selection and response speed as contingency decreases.

In addition to providing a novel test of the Control-Based Response Selection Framework, the study also examined individual differences in the degree to which responses are influenced by action–outcome contingency. Specifically, the impact of contingency on response selection was assessed in relation to four individual difference variables that have previously been associated with the sense of agency and related processes: attributional style (Penton et al. 2014), schizotypy (e.g. Asai and Tanno 2007; Moore et al. 2011), autistic traits (Zalla et al. 2015) and alexithymic traits (Seth 2013). Given their association with sense of agency, and previous reports that contingency impacts upon sense of agency (Moore and Haggard 2008; Sidarus et al. 2013), one might predict that each of these variables will be associated with the effect of contingency on responses.

## Methods

### Participants

Fifty-six participants (Mean age = 23.56 years, SD age = 4.95 years; 34 females) were recruited using university-wide advertisements. Participants were recruited from an opportunity sample: all participants who responded to a recruitment advert within a 6-week time period were tested. All participants had normal or corrected-to-normal vision. The study was approved by King’s College London Psychiatry, Nursing and Midwifery Research Ethics Subcommittee.

### Materials and methods

#### Modified motivation from control task

Participants completed a modified version of the motivation from control task created by Karsh and Eitam (2015b). Participants were instructed to place the index and middle fingers of their left (S, D) and right hands (K, L) on four keys on a keyboard. In this version, participants were cued to the start of a trial by a coloured frame appearing around the perimeter of the screen which remained on screen for 1000 ms. Once this frame appeared, participants were required to press one of the four response keys as randomly as possible. On a given trial, the likelihood of an internally generated event (caused by the participant making a button press) and an externally generated event (outside of the participant’s control) was varied to manipulate action–outcome contingency. The same event (a white dot appearing on the screen for 150 ms) occurred for internal and external causes, and all participants completed all contingency conditions. Following a trial, the coloured frame disappeared and a black screen was presented for 1000 ms. The main dependant variables were response time (calculated as the time between the onset of the frame and button press) and the proportion of key presses for each button. Two factors were manipulated within subjects; the probability of the outcome occurring due to a button press (0%, 30%, 60%, and 90% of key presses) and the probability of the outcome occurring without any button press (0%, 30%, 60%, and 90% of trials). Thus, the following manipulations were introduced.

##### Internally generated event (probability manipulation)

The probability of an internally generated event varied depending on the key pressed. Each key was assigned a different probability of causing an event when pressed (either 0%, 30%, 60% or 90% chance of outcome when pressed) as in the mixed condition of the original study (Karsh and Eitam 2015b). The key assigned to each probability (S, D, K or L) was randomised across participants and remained constant throughout the experiment. Thus, the probability of the participant causing an event when pressing a particular key remained constant throughout the experiment.

##### Externally generated event (contingency manipulation)

Previous paradigms investigating the sense of agency have used external contextual cues (e.g. colour) to manipulate feelings of agency (Aarts et al. 2009). Additionally, research investigating contingency learning demonstrates that contextual cues can be learned by the participant within 40 trials (Cook et al. 2010). Thus, the current experiment employed coloured contextual cues to indicate change in action–outcome contingency throughout the experiment. The likelihood of an externally generated event varied in blocks of 40 trials throughout the experiment. Each context, signalled by a coloured frame (blue, purple, green or orange), was assigned a different probability of an externally generated event occurring on a given trial (0%, 30%, 60%, and 90% chance of outcome occurring on a given trial). The colour assigned to each probability was randomised across participants and remained constant throughout the experiment. The highest action–outcome contingency condition (0% chance of an externally generated event) is similar to the procedure for the mixed-condition group in Karsh and Eitam’s (2015b) study. The order of block type was pseudo-randomised to ensure that two of the same probability blocks were not presented one after another. Participants were not explicitly informed of the association between colour and the probability of externally generated events. In addition, no information was given about the event (appearance of a white dot) prior to completion of the task (as per Karsh and Eitam 2015b; Karsh et al. 2016).

To establish when in time the externally generated events were to be presented on the screen, reaction times from the participant’s button presses in the previous block were recorded. Values falling within the interquartile range were selected and median values were removed. A median split was conducted on the remaining values to split them into fast and slow RTs. 300 ms was subtracted from each of the fast RTs to ensure that externally generated cues presented at these times would occur prior to a participant’s button press (early externally generated event). 100 ms was subtracted from each of the slow RTs to take into account the reduction in RTs associated with task learning and to ensure that these externally generated events would occur shortly after a participant’s button press but still within the trial timeframe (late externally generated event). Thus, in any given block, 50% of externally generated events were designed to occur prior to a participant’s button press and 50% were designed to occur following a participant’s button press.

#### Self-report measures

Participants completed four self-report questionnaires prior to attending the lab-based testing session. These are detailed below:

##### Toronto alexithymia questionnaire (TAS-20)

The TAS-20 is a measure of alexithymia (a sub-clinical trait characterised by a difficulty describing and identifying one’s own emotional state; Bagby et al. 1994). Participants are asked to rate the extent to which they agree or disagree with 20 items using a Likert scale (possible responses are: definitely disagree, slightly disagree, neither agree nor disagree, slightly agree, definitely agree). Responses are scored from 1 to 5 (i.e. a response of “definitely disagree” would score 1 and a response of “definitely agree” would score 5). Each of the 20 items represent 1 of 3 factors: Describing Feelings (e.g. “It is difficult for me to find the right words for my feelings”), Identifying Feelings (e.g. “I am often confused about what emotion I am feeling”), and Externally Oriented Thinking (e.g. “I prefer to just let things happen rather than to understand why they turned out that way”). After accounting for reverse-scored items, scores for each item are summed to create a total score. A cut-off score of 60 is used to indicate high Alexithymic traits.

##### Autism quotient (AQ)

The AQ is a measure of sub-clinical autistic-like traits (Baron-Cohen et al. 2001). Participants are asked to rate the extent to which they agree or disagree with 50 items using a Likert scale (possible responses are: definitely agree, slightly agree, slightly disagree, and definitely disagree). Each of the 50 items represent 1 of 5 factors: Social Skills (e.g. “I find it hard to make new friends”), Attention Switching (e.g. “I frequently get so strongly absorbed in one thing that I lose sight of other things”), Attention to Detail (e.g. “I usually notice car number plates or similar strings of information”), Communication (e.g. “Other people frequently tell me that what I’ve said is impolite, even though I think it is polite”), and Imagination (e.g. “When I’m reading a story, I can easily imagine what the characters might look like”). Responses are summed to calculate an overall score. A cut-off score of 32 is used to indicate high Autistic-like traits.

##### Peters delusion inventory (PDI)

The PDI is a measure of schizotypy (a set of a sub-clinical personality traits related to schizophrenia including delusional ideation, Peters et al. 1999). Participants are asked to answer ‘yes’ or ‘no’ to 21 questions about beliefs and vivid mental experiences (e.g. “Do your thoughts ever feel alien to you in some way?”). If participants agree with the statement they are then required to rate the question along 3, 5-point Likert scales measuring how distressing the event is (distress factor), how often they think about it (preoccupation factor) and how true they think it is (conviction factor). Responses are scored from 1 to 5 with low scores reflective of “not at all” responses (e.g. “not at all distressing”) and high scores reflective of “very” responses (e.g. “very distressing”). Scores from all the subscales are summed to create an overall score. Higher overall scores are indicative of higher levels of delusional ideation.

##### Attributional style questionnaire (ASQ)

The ASQ is a measure of attributional style (the style one uses to explain causality of positive and negative life events, Peterson et al. 1982). Participants are asked to write the most likely cause of 12 (6 positive and 6 negative) hypothetical events (e.g. “A friend compliments you on your appearance”) and then rate the cause along 3, 7-point Likert scales reflecting the extent to which the participant feels the cause of the event was due to an internal factor (the self) or an external factor (internality factor), the stability of the cause of the event across time (stability factor) and the extent to which the cause of the event is unique to one domain or universal (globality factor). A positive attributional style is reflected by responses where participants attribute the cause of positive life events to the self (internality), as stable across time (stability) and present across multiple life domains (globality) and negative life events as the opposite. A negative attributional style is reflected by responses where participants attribute the cause of negative life events to the self (internality), as stable across time (stability) and present across multiple life domains (globality) and positive life events as the opposite. Positive composite scores reflect more positive attributional styles, whereas negative composite scores reflect more negative attributional styles. Scores were summed for positive and negative life events. The negative composite score was then taken from the positive composite score to create an overall score. High scores reflect a more positive attributional style.

### Procedure

Participants completed the questionnaires in a randomised order online prior to attending the lab-based testing session in which they performed the modified motivation from control task. During the computer task, participants were asked to press one of four buttons on every trial. Participants were asked to make these button presses as randomly as possible (to aim for as equal a distribution of button presses as possible, as in Karsh and Eitam’s 2015b study) and as fast as possible following the cue. Participants were presented with 1 practice block of 40 trials to familiarise themselves with the task. The probability of a key press producing an outcome during the practice was equal across the four keys (50% chance of outcome when any key was pressed). The probability of an outcome occurring in the absence of a key press was also set to 50%. The colour frame in the practice trials was different to that of the experimental trials but the white dot event was the same. Following this, participants completed 12 blocks of 40 trials (3 blocks for each contingency condition). Participants had the opportunity for a break at the end of each block. The experiment took approximately 45 min to complete (questionnaires ~ = 20 min, computer task ~ = 25 min). The experimental task was created and presented using Matlab 8.0 (Mathworks) with the Cogent 2000 toolbox (http://www.vislab.ucl.ac.uk/Cogent).

## Results

### Reaction time analysis

Median reaction times for each of the four buttons (0%, 30%, 60% and 90% chance of an internally generated event) were calculated for each of the four levels of contingency (0%, 30%, 60% and 90% chance of an externally generated event). Median RTs were calculated instead of mean RTs for each participant as RTs tend to come from skewed distributions and the median is less sensitive to outlier data (Whelan 2008). Seven participants were removed from analysis for having outlying median reaction times more than 2SD away from the group mean (mean of the medians) in 1 (*n* = 2) or more (*n* = 5) of the conditions. Data from the remaining 49 participants were analysed (*Mean age* = 23.69 years, SD *age* = 4.97 years; *females* = 32). Greenhouse–Geisser corrected significance levels are reported when sphericity assumptions were violated.

#### Effects of probability and contingency

A 4 (internally generated probability [0%, 30%, 60%, 90%]) × 4 (externally generated probability [0%, 30%, 60%, 90%]) repeated measures ANOVA was run on median reaction time data (see Table 1 for a summary of RT data). The main effect of internal probability was not significant (*F*(3,144) = 1.007, *p* = .383, *η*_p_^2^ = 0.021), and neither was the main effect of external probability (*F*(3,144) = 0.578, *p* = .630, *η*_p_^2^ = 0.012). However, there was a significant internal x external interaction (*F*(9,432) = 3.14, *p* = .005, *η*_p_^2^ = 0.061), demonstrating that the effect of internal probability on RTs varied as a result of manipulating external probability (i.e. an effect of contingency).

**Table 1:** Mean of medians and SD (in parentheses) reaction times (ms) for each combination of the internally- and externally generated event conditions

	Probability of internally generated event				
	0%	30%	60%	90%	Overall
Probability of externally generated event					
0%	532.23 (51.61)	527.03 (48.05)	523.52 (49.84)	521.82 (52.39)	526.15 (50.47)
30%	518.33 (62.04)	521.32 (64.97)	531.85 (68.42)	523.31 (63.29)	523.70 (64.68)
60%	526.07 (60.96)	523.71 (58.19)	518.16 (51.93)	527.95 (61.34)	523.97 (58.11)
90%	521.08 (67.64)	513.52 (67.37)	524.79 (64.54)	517.87 (66.55)	519.32 (66.53)
Overall	524.43 (60.56)	521.40 (59.65)	524.58 (58.68)	522.74 (60.89)	

One-way ANOVAs revealed a significant effect of internal probability on RTs when action–outcome contingency was higher (0% chance of an external flash [*F*(3,144) = 3.72, *p* = .019, *η*_p_^2^ = 0.072], 30% chance of an external flash [*F*(3,144) = 2.87, *p* = .038, *η*_p_^2^ = 0.057]) but no significant effect of probability when action–outcome contingency was lower (60% chance of an external flash [*F*(3,144) = 1.31, *p* = .272, *η*_p_^2^ = 0.027], 90% chance of an external flash [*F*(3,144) = 1.87, *p* = .137, *η*_p_^2^ = 0.038]).

#### Individual differences in the influence of contingency

The relationship between individual differences in alexithymia, autistic traits, schizotypy and attributional style, and the extent to which speed of participants’ actions was influenced by action–outcome contingency, was also examined. To do this, a single value was calculated to reflect the influence of contingency on RTs. Specifically, for each participant, for the first level of contingency (0% likelihood of an external flash), median RTs from each button (buttons with 0%, 30%, 60%, and 90% chance of an internally generated flash) were multiplied by relative weightings (− 1.5, − 0.5, 0.5 and 1.5) and then summed to create an overall score of the influence of probability in the highest action–outcome contingency condition. This was repeated for the other three levels of contingency (30%, 60%, and 90% chance of an external flash) resulting in four scores reflecting the influence of probability on RTs at each level of contingency. The summed scores (one from each contingency level) were multiplied by the same weightings (− 1.5, − 0.5, 0.5 and 1.5 for 90%, 30%, 60% and 0% external probability conditions) to calculate an overall score. Higher scores reflect an influence of contingency on RT by demonstrating a reduction in the influence of internal probability with decreasing contingency. These overall contingency scores were then standardised by dividing them by the participant’s mean RT.

Given that this analysis was concerned with the overall influence of contingency, a new outlier analysis was conducted on composite scores for all 56 participants. This revealed four participants who had a composite score greater than two SD from the mean in any one of the four contingency conditions, who were subsequently excluded from the analysis. Data from the remaining 52 participants were included in the analysis (*Mean age* = 23.69 years, SD *age* = 5.09 years; *females* = 32). Of these, only 51 participants completed the self-report measures for alexithymia, autistic traits and attributional style. All 52 participants completed the self-report measure for schizotypy.

**Fig. 1:**
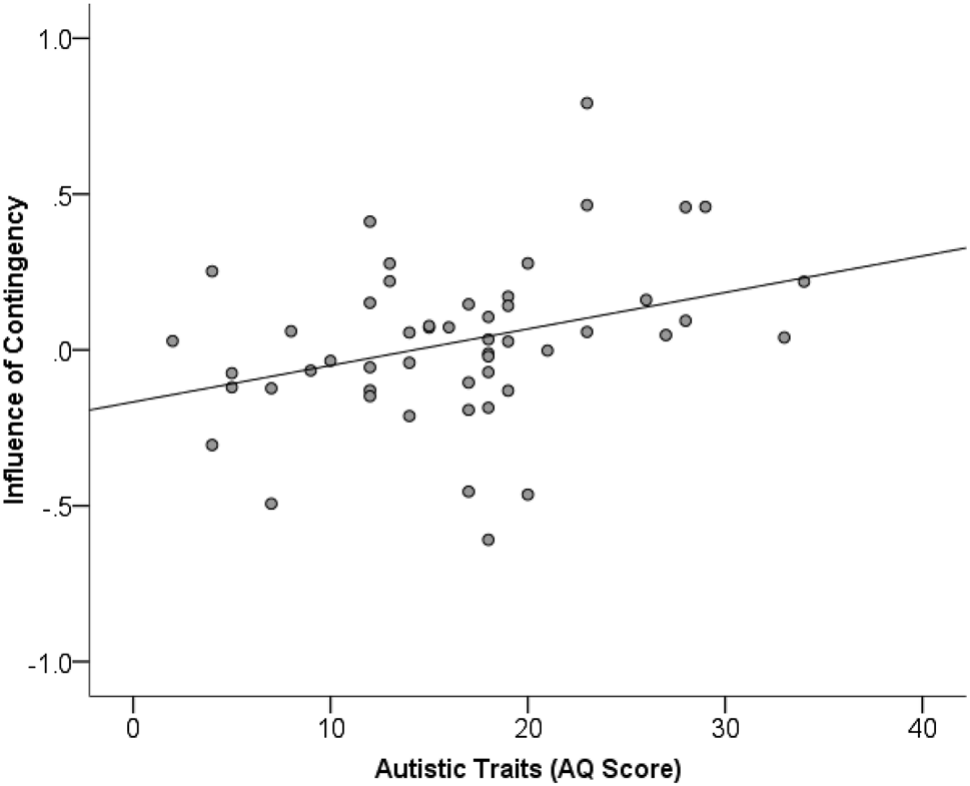
Relationship between influence of contingency on RTs and AQ score. Increasing autistic traits are correlated with a greater influence of contingency on RTs. Higher influence of contingency scores demonstrates a reduction in the influence of the probability of a keypress-related outcome with decreasing contingency

Correlational analyses were then used to investigate the relationship between the overall contingency score and alexithymia (*Mean* = 45.92, SD = 12.41), autistic traits (*Mean* = 16.47, SD = 7.35), schizotypy (*Mean* = 61.54, SD = 43.10) and attributional style (*Mean* = 0.74, SD = 1.21). This revealed a positive correlation between influence of contingency and autistic traits (*r*(49) = 0.338, *p* = .015) reflective of individuals with higher autistic traits showing a greater influence of contingency on RTs (see Fig. 1). There was also a positive correlation between alexithymic traits and influence of contingency (*r*(49) = 0.304, *p* = .030). It should be acknowledged, however, that neither of these correlations survived correction for multiple comparisons (alpha of 0.0125), although the correlation between autistic traits and influence of contingency approached this level. There was no significant relationship between influence of contingency and schizotypal traits (*r*(50) = 0.113, *p* = .425), or attributional style (*r*(49) = − 0.057, *p* = .690).

### Frequency analysis

Frequency of button presses for each of the four buttons (0, 30, 60 and 90% chance of an internally generated event) at each of the four levels of contingency (0, 30, 60 and 90% chance of an externally generated event) was determined by calculating the percentage of overall presses for each button at each level of contingency. Nine participants were removed from analysis for having outlier data more than two SD away from the mean in 1 (*n* = 4) or more (*n* = 5) of the conditions. Data from the remaining 47 participants were analysed (*Mean* age = 23.61 years, SD *age* = 4.96 years; *females* = 29).

#### Effects of probability and contingency

Due to the non-independence of the frequency data, they are unsuitable for analysis with ANOVA. Previous papers investigating the effect of probability on response frequencies have modelled the data using Dirichlet distributions. This approach was adopted here to examine the influence of probability at each of the four levels of contingency. Such models were chosen as they allow for the analysis of related random probability distributions (see Buis et al. 2010). Specifically, the Dirichlet distribution takes into account that pressing one of the four buttons reduces the number of remaining button presses in the experiment, and thus impacts the frequency distributions for the other three buttons. Four models were calculated for button presses at each level of contingency to investigate the extent to which buttons with a higher probability of outcome (30, 60 and 90% chance of outcome when pressed) were preferred to buttons with zero probability of an outcome (see Table 2 for a summary of frequency data). Specifically, for each of the four levels of contingency, the logarithm of the ratio of the proportion of presses for each of the three keys associated with an outcome (30%, 60%, and 90% chance of outcome) and the proportion of presses for the key associated with 0% chance of an outcome were modelled using Dirichlet distributions (for similar analyses see Karsh and Eitam 2015b; Karsh et al. 2016; Eitam et al. 2013). A significant effect of probability was not observed for any of the 4 levels of contingency (0% chance of external outcome, *χ*^2^ = 4.82, *p* = .185; 30% chance of external outcome, *χ*^2^ = 6.14, *p* = .105; 60% chance of external outcome, *χ*^2^ = 6.27, *p* = .099; 90% chance of external outcome, *χ*^2^ = 4.67, *p* = .198). To examine whether a main effect of probability was present after accounting for the contingency manipulation, another model was calculated as above but which included contingency as a covariate. Testing for the main effect of internal probability when including the probability of an externally generated outcome as a covariate revealed a significant main effect of internal probability (*χ*^2^ = 8.42, *p* = .038). This reflected a positive linear relationship between probability of outcome for a given button with a non-zero probability of outcome, and proportion of presses for a given button with a non-zero probability of outcome (i.e. buttons with higher probabilities of outcome were pressed more frequently). This demonstrates that when removing variance associated with the contingency manipulation, probability of an internally generated outcome influences frequency of button presses (consistent with work by Karsh and Eitam 2015b; Karsh et al. 2016; Eitam et al. 2013).

**Table 2:** Mean and SD (in parentheses) percentages of responses for keys associated with each probability of an internally generated event at each level of externally generated event probability

	Probability of internally generated event				
	0%	30%	60%	90%	Overall
Probability of externally generated event					
0%	24.56 (4.70)	23.34 (4.27)	26.02 (4.59)	26.09 (7.41)	25 (5.24)
30%	24.25 (4.26)	23.78 (4.85)	25.68 (4.43)	26.29 (5.85)	25 (4.85)
60%	24.57 (3.98)	23.66 (3.87)	25.56 (3.59)	26.22 (5.11)	25 (4.14)
90%	25.52 (4.64)	23.48 (4.60)	25.56 (4.17)	25.64 (6.04)	25 (4.86)
Overall	24.73 (4.40)	23.57 (4.40)	25.71 (4.20)	26.06 (6.10)	

This analysis does not allow for an effect of contingency to be identified, however, and so to test for an effect of contingency the same approach was used as in the individual differences analysis of the RT data. A single value was calculated to reflect the change in the effect of internal probability on frequency of responses as a function of external probability of the outcome (i.e. contingency). Specifically, for the first level of contingency (0% likelihood of an external flash), percentage of responses for each button (buttons with 0%, 30%, 60%, and 90% chance of an internally generated flash when pressed) was multiplied by relative weightings (− 1.5, − 0.5, 0.5 and 1.5) and then summed to create an overall score of the influence of probability in the highest action–outcome contingency condition. This was repeated for the other three levels of contingency (30%, 60%, and 90% chance of an external flash on a given trial) resulting in four scores reflecting the influence of probability on pattern of response frequencies at each level of contingency. The summed scores (one from each contingency level) were multiplied by the same weightings (− 1.5, − 0.5, 0.5 and 1.5 for 90%, 30%, 60% and 0% external probability conditions) to calculate an overall score. As with the RT analysis, a new outlier analysis was conducted on these contingency composite scores for all 56 participants. This revealed two participants who had a composite score greater than two SD from the mean in any one of the four contingency conditions, who were subsequently excluded from the analysis. Data from the remaining 54 participants were included in the analysis (*Mean age* = 23.66, SD *age* = 5; 34 *females*). Thus, higher scores reflect an influence of contingency on frequency by demonstrating a reduction in the influence of internal probability with decreasing contingency. These scores were analysed using a one-sample *t* test against zero which was not significant *t*(53) = 0.68, *p* = .50. Thus, this analysis provided no evidence for an effect of contingency on response selection.

#### Individual differences in the influence of contingency

The relationship between individual differences in alexithymia, autistic traits, schizotypy and attributional style and the extent to which frequency of participants’ button selections was influenced by action–outcome contingency, was also examined using the contingency single scores calculated above. Of the 54 participants included in this analysis, 53 participants completed the self-report measures for alexithymia, autistic traits and attributional style. All 54 participants completed the self-report measure for schizotypy.

Correlational analyses were then used to investigate the relationship between the contingency composite scores and alexithymia (*Mean* = 46.38, SD = 11.86), autistic traits (*Mean* = 16.98, SD = 7.38), schizotypy (*Mean* = 61.94, SD = 42.00) and attributional style (*Mean* = 0.70, SD = 1.19). No significant relationship was observed between contingency score and autistic traits (*r*(51) = 0.070, *p* = .618), schizotypal traits (*r*(52) = 0.127, *p* = .360), attributional style (*r*(51) = 0.006, *p* = .968) or alexithymia (*r*(51) = 0.090, *p* = .522).

## Discussion

This study provided a new test of the Control-Based Response Selection Framework (Karsh and Eitam 2015a) within the domain of action–outcome contingency. The contingency between an action (key press) and an outcome (white dot) was varied systematically over experimental blocks such that in some blocks the outcome only occurred in response to an action, while in others the outcome could also occur in the absence of an action. In addition, individual difference factors previously associated with the sense of agency were investigated to determine whether they were associated with the degree to which responses were affected by contingency.

Results with respect to response speed were in accordance with predictions from the Control-Based Response Selection Framework. When contingency was high, reaction times were governed by the probability of the outcome given the action. As contingency decreased (i.e. the probability of an outcome given no action increased), response speed was no longer governed by the outcome probability associated with each key press. In contrast to response speed, response selection (i.e. frequency of specific key presses) was not significantly influenced by action–outcome contingency (in that the pattern of button presses did not differ across contingency conditions) but was influenced by action–outcome probability. These response selection results are therefore consistent with previous research showing an effect of outcome probability on response selection (Karsh and Eitam 2015b; Karsh et al. 2016; Eitam et al. 2013). The response selection results are also consistent with predictions from the Control-Based Response Selection Framework if the contingency manipulation affects implicit, but not explicit sense of agency. Under this framework, actions associated with an implicit sense of agency affect response speed, while those associated with an explicit sense of agency impact both response selection and response speed. This leads to the clear prediction that explicit judgments of agency should not be affected by the contingency manipulation used in the current experiment. While this prediction remains to be tested, previous work has established that manipulation of action–outcome contingency affects implicit sense of agency. For example, Moore et al. (2009) have shown that increased action–outcome contingency compresses the perceived temporal delay between action and outcome (a phenomenon known as intentional binding thought to index implicit feelings of agency). In addition, cues to agency can differentially impact implicit or explicit sense of agency (e.g. Ebert and Wegner 2010) and different mechanisms are thought to underlie these processes (David et al. 2008; Dewey and Knoblich 2014). As such, the current finding that action–outcome contingency affects one index of motivation to act (response speed), but not another (response selection), may provide additional insight into a growing literature on differential processes involved in the experience of implicit and explicit sense of agency.

Consideration of the possible differential effects of implicit and explicit sense of agency highlights a limitation of the current study; the mediating effect of the sense of agency between modulation of action–outcome contingency and response speed was inferred but not tested. The addition of explicit or implicit tests of the impact of this contingency manipulation on sense of agency would have enabled the mediating role of the sense of agency to be tested rather than assumed. Previous studies have shown a relationship between control-based response selection and explicit sense of agency (Karsh and Eitam 2015b). It should be noted, however, that in these studies participants were asked about their sense of agency at the end of the response selection task. The inclusion of such measures in the current paradigm would have interfered with the main aim of the experiment, which was to determine whether response selection and/or speed would be affected by a contingency manipulation that was varied across blocks.

Four individual difference factors were measured to investigate whether they were associated with the impact of contingency on response selection or response speed. Both the degree of alexithymic and autistic traits were associated with the impact of contingency on response speed, although after correction for multiple comparisons, only the effect of autistic traits approached significance. The association between increasing autistic traits and an increasing effect of action–outcome contingency may reflect a tendency for individuals with lower autistic traits to experience a greater sense of agency, and therefore increased motivation to act in accordance with their perceived agency, over events which are in fact out of their control. Research has demonstrated that a sense of agency can be experienced even in the absence of an action (Wegner 2004; Moore and Haggard 2008), thus the differences observed here between individuals with higher and lower autistic traits may reflect a bias toward (incorrectly) labelling the cause of an event as internal in individuals with lower autistic traits. More generally, this may point to individual differences in the extent to which participants privilege internal (e.g. intention, prediction) and external (e.g. outcome characteristics) cues to agency. It has been suggested that individuals with autism are more likely to rely upon external stimulus-driven information compared to neurotypical controls, who attach a greater weight to prior predictions/expectations (Pellicano and Burr 2012). In the context of the sense of agency, individuals with autism may weight prospective cues to agency (e.g. intention, prediction) less than neurotypical controls, whilst processing of retrospective cues (stimulus properties) remains typical, or is even more accurate than in controls (Zalla and Sperduti 2015). With respect to the current findings, it may be that individuals with high autistic traits are less likely to experience a false sense of agency over externally generated events, as internal cues related to intention may be weighted less heavily than external cues related to the sensory experience of pushing the button and perceiving the outcome. It should be noted however, that under the Control-Based Response Selection Framework the predictive ability of autistic traits could arise from an association between autistic traits, and (1) the ability to determine contingency, (2) the degree to which action–outcome contingency impacts sense of agency, and/or (3), the degree to which sense of agency influences response speed. Again, the inclusion of measures of sense of agency in future work, using a paradigm in which agency can be determined without removing the effect of sense of agency on response selection, would allow these possibilities to be tested.

In summary, the current findings replicate and extend the findings of Karsh and Eitam (2015b) by highlighting the influence of action–outcome contingency on response speed, and add to a growing body of research by Eitam and colleagues showing that multiple cues affecting sense of agency also affect response selection and/or response speed (e.g. temporal contiguity, spatial contiguity, probability; Karsh et al. 2016; Eitam et al. 2013). Results are in accordance with the Control-Based Response Selection Framework (Karsh and Eitam 2015a), suggesting that individuals are motivated to perform actions that afford a sense of agency. They also highlight the fact that individuals higher in autistic traits may have a more veridical understanding of agency, and/or find those actions with high action–outcome contingency especially motivating.

## Data Availability

According to UK research councils' Common Principles on Data Policy, all data supporting this study will be available at: https://osf.io/9hw8m.
